# Supplementary figures and images for: A non-canonical role for the autophagy machinery in anti-retroviral signaling mediated by TRIM5α
Source: PLoS Pathog. 2020 Oct 14;16(10):e1009017. doi: 10.1371/journal.ppat.1009017 (PMC7588057; doi:10.1371/journal.ppat.1009017)

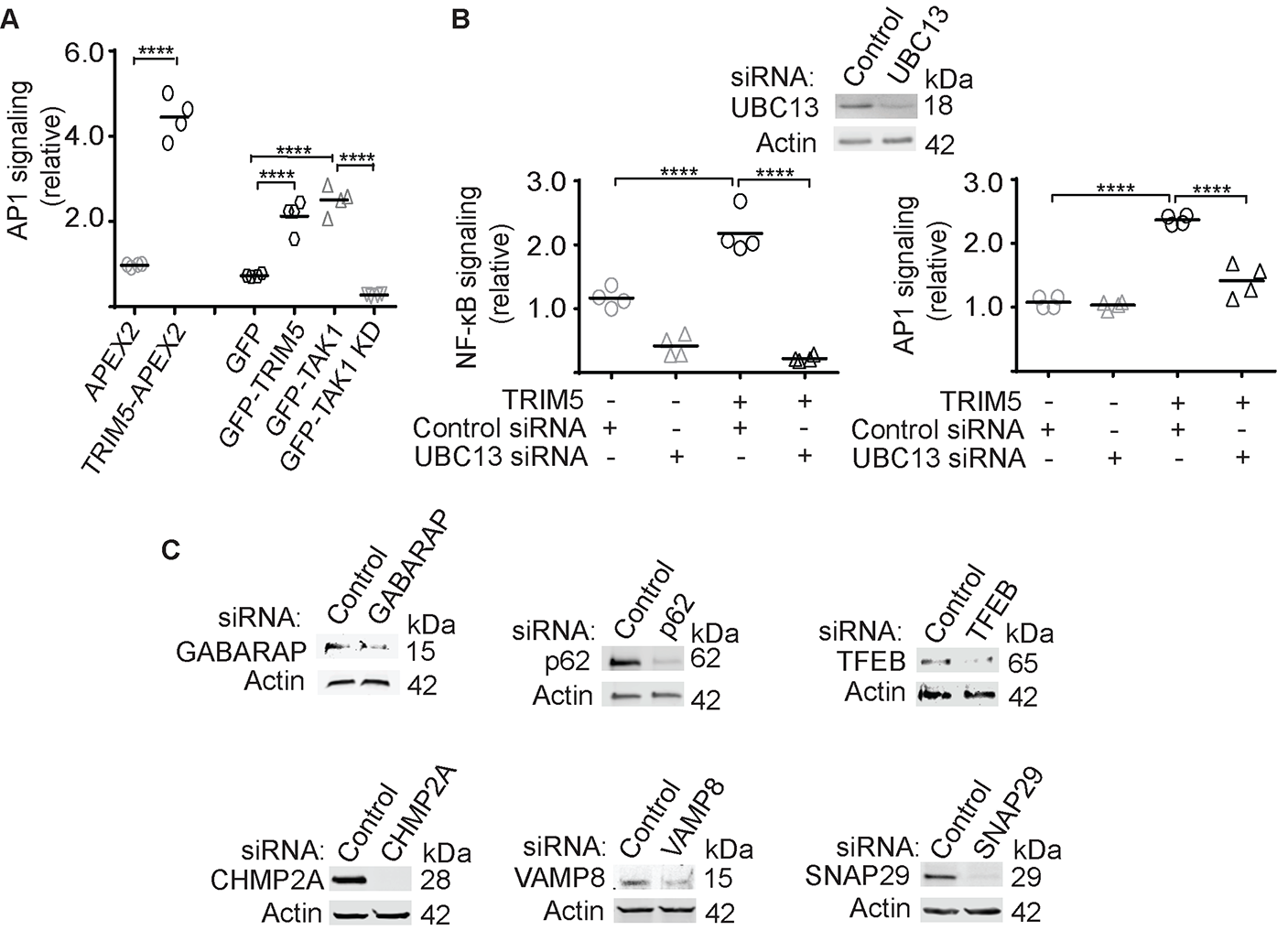

Supplement: S1 Fig — (A) The impact of GFP-TRIM5, TRIM5-APEX2, wt GFP-TAK1 and kinase-dead GFP-TAK1 (K63W) relative to GFP or APEX2 alone on AP1 activity using a dual-luciferase reporter system. (B) Dual-luciferase reporter-based assays determining the effects of siRNA-mediated knockdown of UBC13 on the ability of TRIM5 expression to drive activation of NF-κB or AP1. Immunoblots illustrate knockdown efficiency. (C) Immunoblots showing knockdown efficiency corresponding to the data sets shown in Fig 3A and 3E. Data,****, P < 0.0001 by ANOVA. (TIF) [file ppat.1009017.s001.tif]

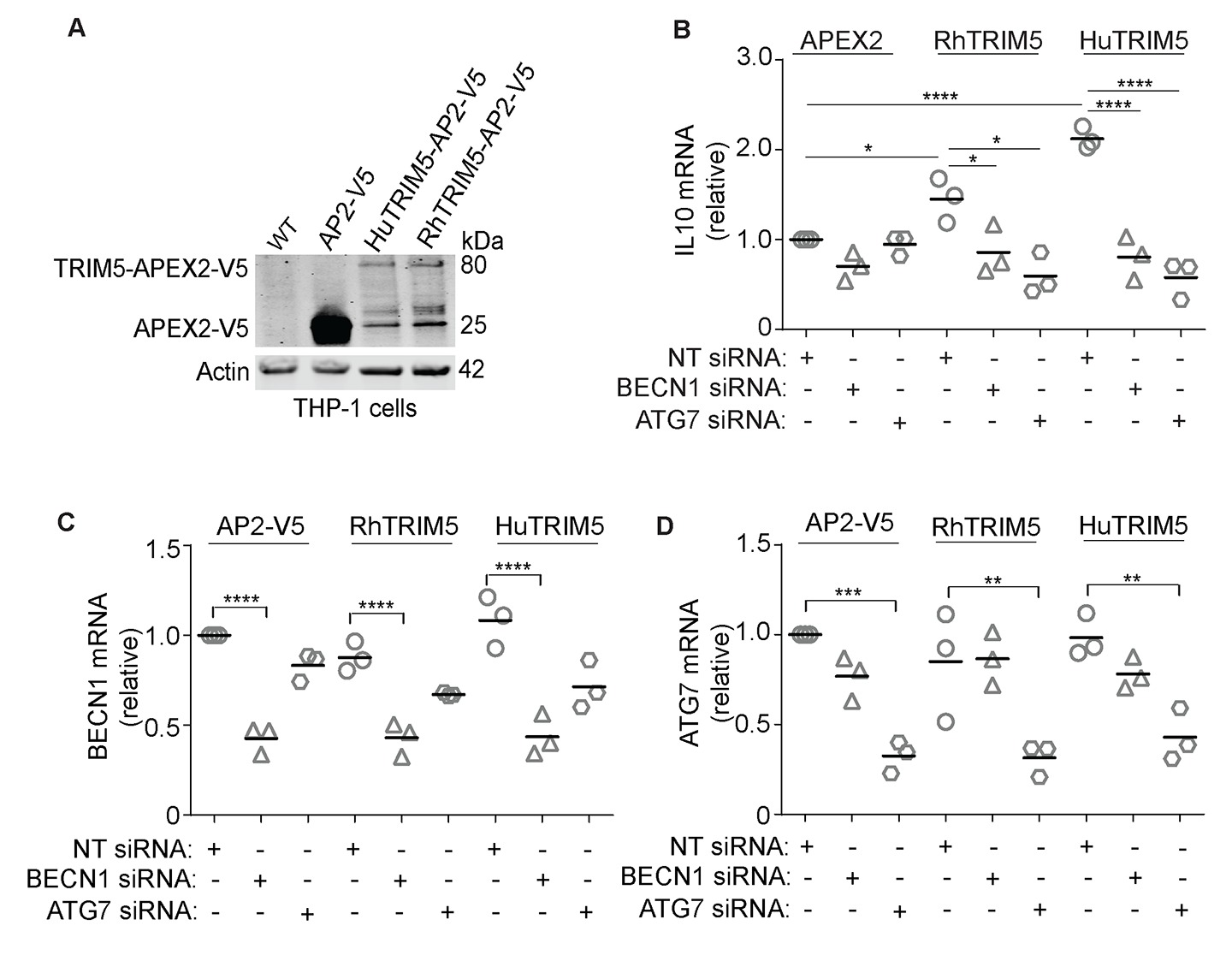

Supplement: S2 Fig — (A) Immunoblot analysis of THP-1 cells stably transduced with lentiviruses expressing APEX-V5 alone (AP2-V5) or TRIM5-APEX2-V5 of human or rhesus origin. Lysates were harvested from THP-1 cells following culture in media containing selective antibiotic, and immunoblots were probed with anti-V5 or anti-actin. (B) Quantitative RT-PCR analysis of IL-10 expression in THP-1 macrophages stably transduced as indicated prior to siRNA-mediated knockdowns of BECN1 or ATG7. NT, non-targeting siRNA. (C,D) Quantitative RT-PCR analysis of knockdown efficiency in differentiated THP-1 cells corresponding to the data shown in (B) and in Fig 2D and 2F. N = 3 independent experiments. Data, *, P < 0.05; **, P < 0.01; ***, P < 0.001, **** by ANOVA. (TIF) [file ppat.1009017.s002.tif]

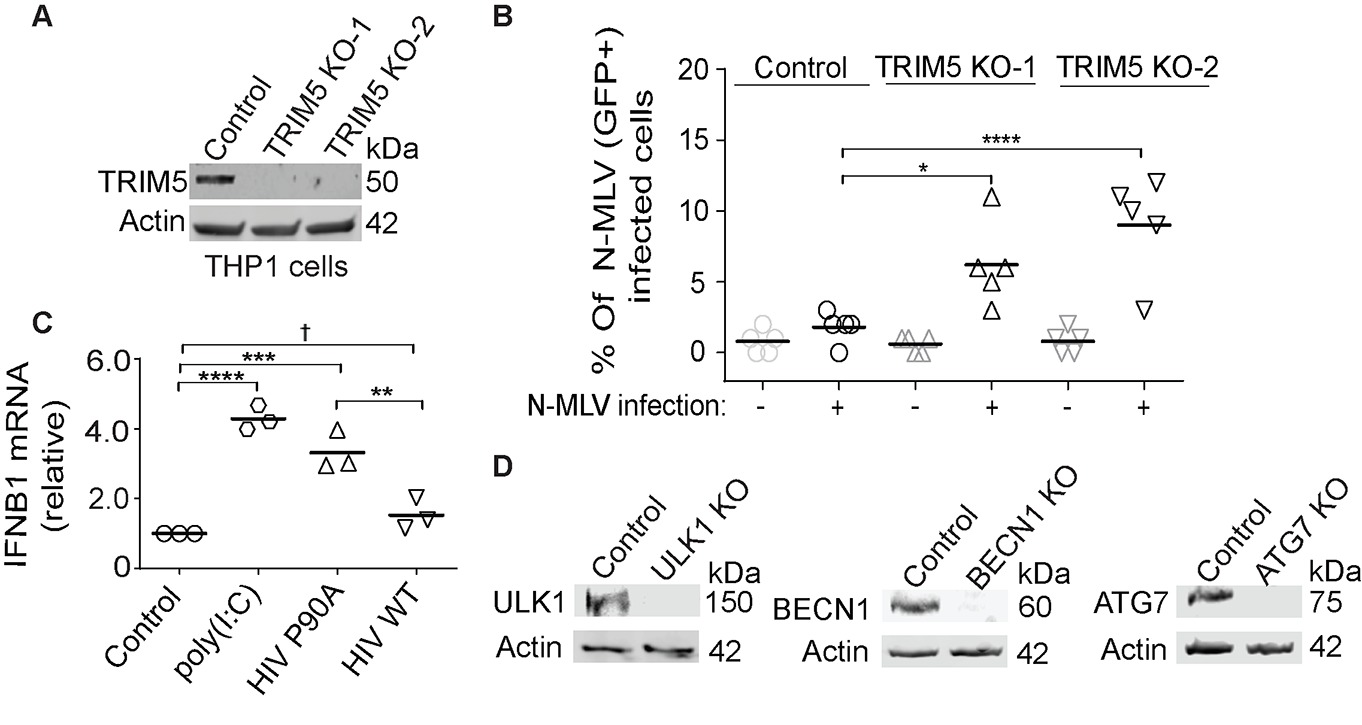

Supplement: S3 Fig — (A) Immunoblot analysis of TRIM5 expression in THP-1 cells transduced with lentivirus encoding Cas9 and either non-targeting (control) or two different TRIM5-targetted guide RNAs. (B) High content imaging-based analysis of GFP-expressing N-MLV infection of control and TRIM5 knockout THP-1 macrophages. (C) Quantitative RT PCR analysis of interferon β mRNA in wild type THP-1 macrophages 2 hours after infection with VSV-G pseudotyped HIV-1 (WT or P90A capsid), both used at a CrFK MOI of 3. TLR-3 ligand poly(I:C) was used as a positive control. (D) Immunoblot analysis of autophagy-factor knockout THP-1 cells generated by transduction with a Cas9/guide RNA expressing lentiviral vector. Data, *, P < 0.05; **, P < 0.01; ***, P < 0.001; ****, P < 0.0001; †, not significant by ANOVA. (TIF) [file ppat.1009017.s003.tif]

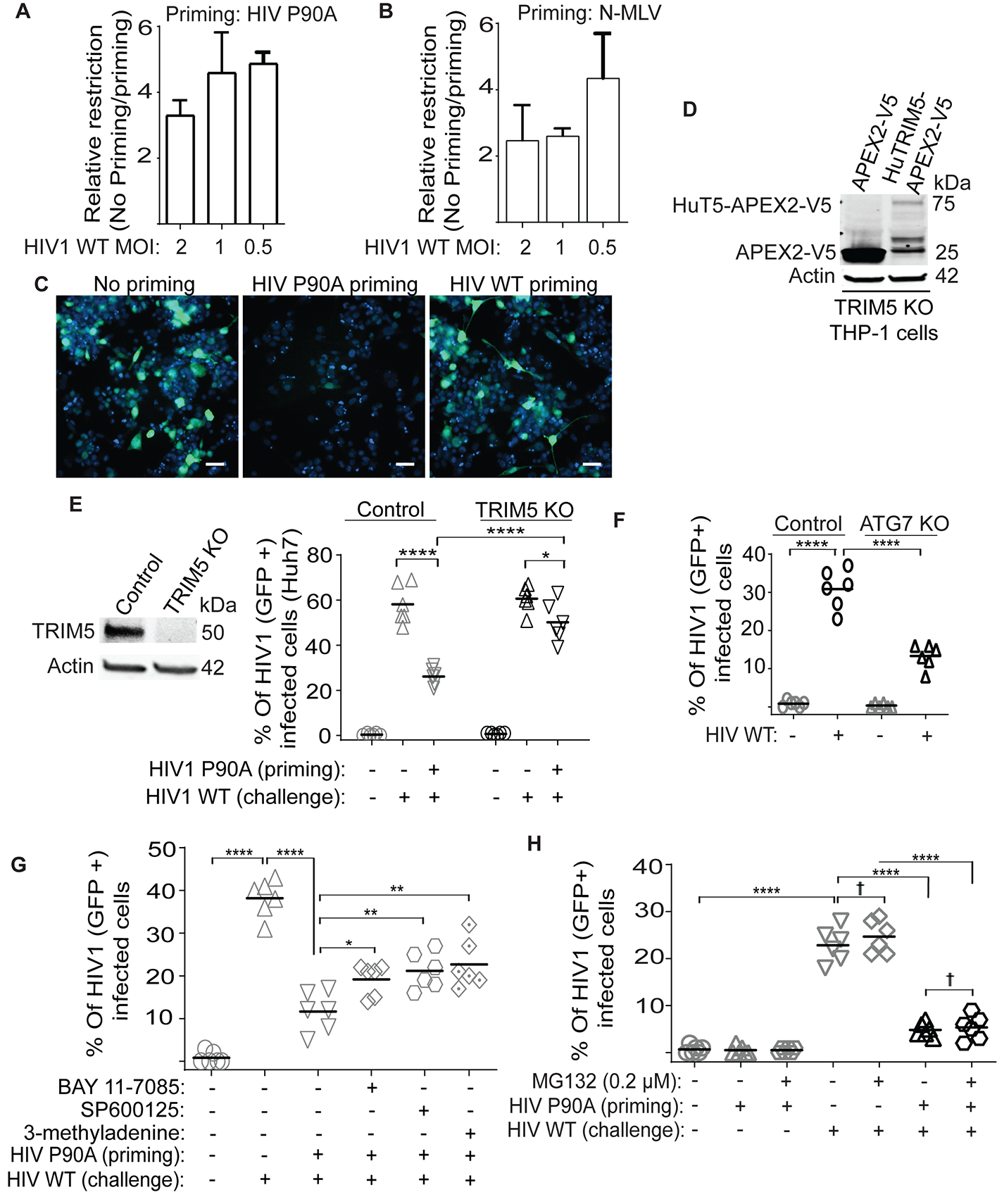

Supplement: S4 Fig — (A) The relative restriction of the challenge virus imposed by prior infection is calculated by dividing the percent of infected cells without priming by the percent of cells after priming. Data, mean + S.E.M; N = 3 experiments as shown in Fig 6C in which cells were primed with HIV-1 CA P90A (CrFK MOI 3) and challenged with different dilutions of WT HIV-GFP. (B) The relative restriction of HIV-GFP following priming (or not) with N-MLV. Data, mean + S.E.M; N = 3 experiments as shown in Fig 6D. Cells were primed with a CrFK MOI of 0.5. (C) The impact of priming with TRIM5-restricted (P90A) or TRIM5-resistant (WT) HIV-1 capsids corresponding to data shown in Fig 6E. Images show representative micrographs from high content imager showing the impacts of priming on HIV-GFP transduction efficiency. Scale bar, 50 μm. (D) Immunoblot analysis of TRIM5 knock-out THP-1 cells transduced with lentivirus expressing APEX2-V5 alone or human TRIM5-APEX2-V5 corresponding to Fig 6G. (E) STR in Huh7 hepatoma cell line. Immunoblot shows TRIM5 knockout efficiency in Huh7 cells. Plot (right), control or TRIM5 KO Huh7 cells were exposed or not to VSV-G pseudotyped HIV-1 P90A (CrFK MOI = 0.25) 24 h prior to being challenged by GFP-expressing VSV-G pseudotyped WT HIV-GFP (CrFK MOI = 0.5). Two days later, the percentage of cells showing green fluorescence was determined by high content imaging. (F) High content imaging-based analysis of WT HIV-GFP infection of control and ATG7 knockout THP-1 cells without priming. Data shown, 1 of 3 experiments. Each data point represents one well of a 96 well plate with >2000 cells analyzed per well. (G) The effect of NF-κB, AP1, and autophagy inhibitory compounds on STR in THP-1 macrophages corresponding to data shown in Fig 7D. Each data point represents one well of a 96 well plate with >2000 cells analyzed per well by high content imaging. (H) The effect of proteasome inhibition with MG132 on the ability of HIV-1 CA P90A priming on WT HIV-GFP infection. Ce [file ppat.1009017.s004.tif]

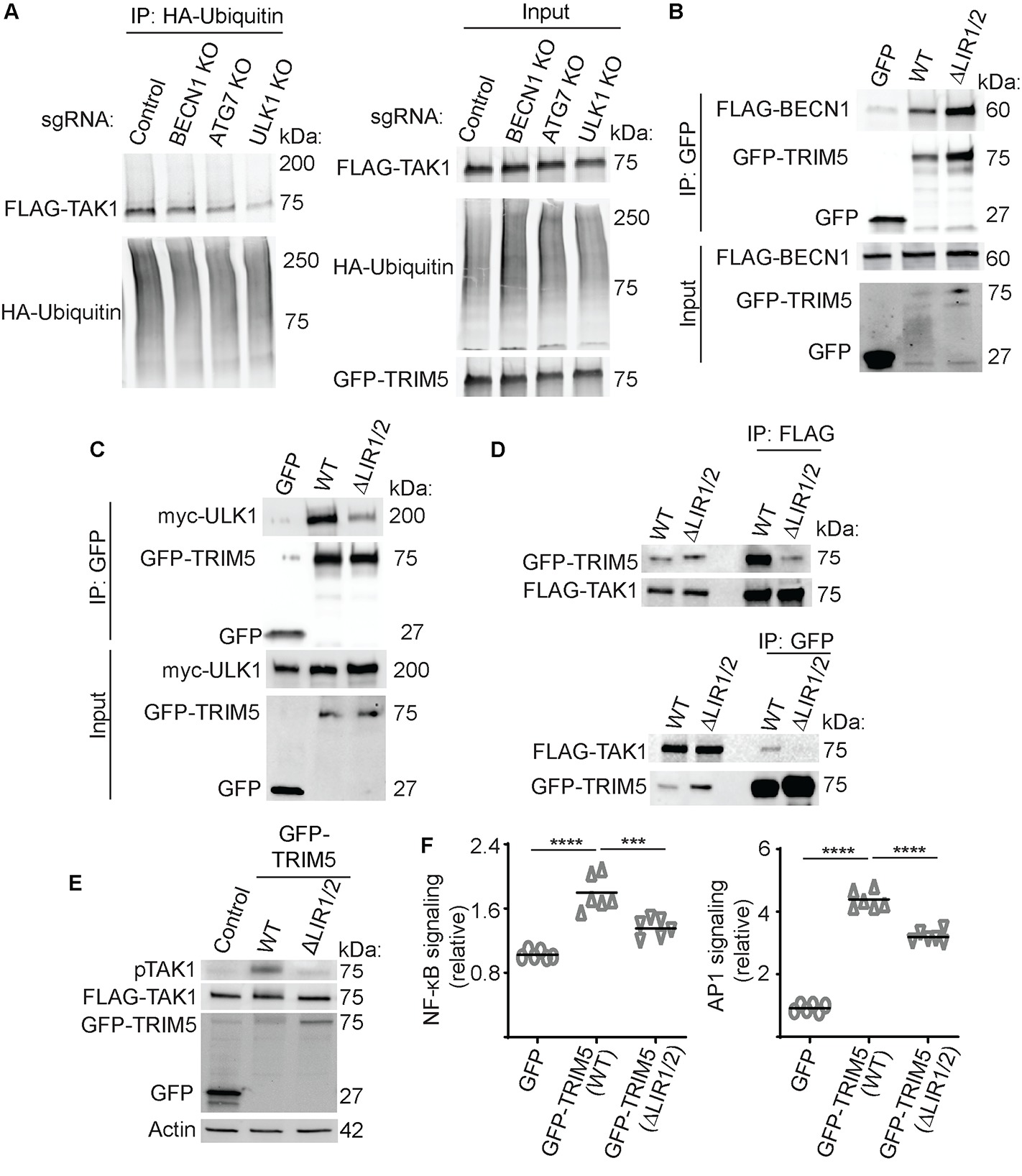

Supplement: S5 Fig — (A) Co-immunoprecipitation analysis of the interactions between HA-ubiquitin and FLAG-TAK1 in WT or autophagy factor knockout cells expressing GFP-TRIM5. (B,C) Co-immunoprecipitation analysis of interactions between GFP-tagged WT and ΔLIR1/2 mutant (F187A, L190A, W196A, E197A) TRIM5 and FLAG-BECN1 (B) or Myc-ULK1 (C) from transiently transfected HEK293T cell lysates. Anti-GFP was used for pull-down. (D) Co-immunoprecipitation analysis of interactions between GFP-tagged WT or ΔLIR1/2 TRIM5 and FLAG-TAK1 from lysates of transiently transfected HEK293T cells subjected to immunoprecipitation with anti-FLAG (top) or anti-GFP (bottom) with immunoblots probed as indicated. (E) The effect of WT or ΔLIR1/2 GFP-TRIM5 on the abundance of active phospho-TAK1 (phospho-Thr184/187) in transiently transfected HEK293T cell lysates. (F) The impact of ΔLIR1/2 mutation on the ability of GFP-TRIM5 to induce NF-κB and AP1 activation as measured by luciferase reporters in HEK293T cells. Each data point represents an independent biological replicate, ***, P < 0.001; ****, P < 0.0001 by ANOVA. (TIF) [file ppat.1009017.s005.tif]
