# Supplementary material for: A non-canonical role for the autophagy machinery in anti-retroviral signaling mediated by TRIM5α
Source: PLoS Pathog. 2020 Oct 14;16(10):e1009017. doi: 10.1371/journal.ppat.1009017 (PMC7588057; doi:10.1371/journal.ppat.1009017)
Supplement: Attachment — Submitted filename: Response to reviewers.pdf [file ppat.1009017.s006.pdf]

Dear Dr. Mandell,

Thank you very much for submitting your manuscript "A non-canonical role for the autophagy machinery in anti-retroviral signaling mediated by TRIM5α." for consideration at PLOS Pathogens. As with all papers reviewed by the journal, your manuscript was reviewed by members of the editorial board and by several independent reviewers. In light of the reviews (below this email), we would like to invite the resubmission of a significantly-revised version that takes into account the reviewers' comments. In particular, we would need to see a meaningful response to the comments of reviewer 1 and some rewriting of the results section in response to reviewer 2.

We cannot make any decision about publication until we have seen the revised manuscript and your response to the reviewers' comments. Your revised manuscript is also likely to be sent to reviewers for further evaluation.

When you are ready to resubmit, please upload the following:

[1] A letter containing a detailed list of your responses to the review comments and a description of the changes you have made in the manuscript. Please note while forming your response, if your article is accepted, you may have the opportunity to make the peer review history publicly available. The record will include editor decision letters (with reviews) and your responses to reviewer comments. If eligible, we will contact you to opt in or out.

[2] Two versions of the revised manuscript: one with either highlights or tracked changes denoting where the text has been changed; the other a clean version (uploaded as the manuscript file).

Important additional instructions are given below your reviewer comments.

Please prepare and submit your revised manuscript within 60 days. If you anticipate any delay, please let us know the expected resubmission date by replying to this email. Please note that revised manuscripts received after the 60-day due date may require evaluation and peer review similar to newly submitted manuscripts.

Thank you again for your submission. We hope that our editorial process has been constructive so far, and we welcome your feedback at any time. Please don't hesitate to contact us if you have any questions or comments.

Sincerely,

Bryan R. Cullen
Associate Editor
PLOS Pathogens

Susan Ross
Section Editor

PLOS Pathogens

Kasturi Haldar
Editor-in-Chief
PLOS Pathogens
orcid.org/0000-0001-5065-158X

Michael Malim
Editor-in-Chief
PLOS Pathogens
orcid.org/0000-0002-7699-2064

****

Reviewer's Responses to Questions

Part I - Summary
Please use this section to discuss strengths/weaknesses of study, novelty/significance, general execution and scholarship.

Reviewer #1: TRIM5a is one of the best-studied Tripartite motif proteins. It is well-recognized as a Lentiviral restriction factor. While it is known that TRIM5a recognizes capsids in a species-specific manner, and induces premature uncoating, the consequences and mode of signaling induced by TRIM5a are less clear. Particularly the involvement of autophagy has been under debate.
It has been proposed that TRIM5a may serves as an innate immune receptor. Although the pattern recognized by this factor may be really specialized to qualify as a pattern recognition receptor.
In this manuscript Saha and co-workers show that TRIM5-dependent cytokine/Nf-kB induction requires core autophagy proteins such as ATG7 or beclin-1. Pre-stimulation of cells with TRIM5a-recognized capsid renders the cells less susceptible towards a subsequent HIV-1 infection.
Most experiments are well conducted and controlled. The findings presented would be novel and an interesting aspect of signaling regulation by autophagy. However, there are a few things which may need some clarification.

Reviewer #2: This paper describes a non-canonical role for the cellular autophagy machinery in TRIM5 mediated innate signalling. I think this is a very important result. However, the results section of the paper is at times rather hard to follow with some data seemingly randomly assigned to Figs or Suppl Figs- did a limit in the number of figures play a role? Why for example did parts A and B of Fig 2 appear there when the experiments reported were performed in HEK cells, not macrophages as in the figure title. I wonder whether some of the normalized data would be better presented as tables. I would also note that improved quantification would be very helpful in assessing many of the protein blots.

Reviewer #3: This is an nice study that is comprehensive and well controlled. The paper is well-written and makes important conceptual advances in the field of antiviral signaling pathways, by enhancing our understanding of how autophagy contributes to the ability of TRIM5a to promote the IFN response.
In my opinion, this work will be of general interest to a broad audience in molecular biology and cell biology.

We sincerely thank all reviewers for their comments and suggestions. As detailed below, we have made substantial revisions to our manuscript in response to their comments. This includes providing new data sets and quantitative analysis of the data. We also have substantially rearranged the presentation of the results and have moved several data panels out of the supplementary figures and into main figures. Finally, we have provided a new graphical representation of our findings to help explain our model.

Part II – Major Issues: Key Experiments Required for Acceptance
Please use this section to detail the key new experiments or modifications of existing experiments that should be absolutely required to validate study conclusions.

Generally, there should be no more than 3 such required experiments or major modifications for a "Major Revision" recommendation. If more than 3 experiments are necessary to validate the study conclusions, then you are encouraged to recommend "Reject".

Reviewer #1: - There is a control missing in P90A capsid pre-stimulation assay. How do capsids modulate the IFN-b response and a subsequent infection that are not recognized by TRIM5a? Please include priming with a non TRIM5-sensitive virus in Fig. 4.

Our data (originally Figure 4E and S4C, now Figure 6E and S4C in the revised submission) demonstrate that exposure to WT HIV (not sensitive to human TRIM5) failed to 'prime' THP-1 macrophage-like cells against subsequent HIV experiment. We apologize for insufficiently highlighting this important control.

Our model for how this works is that TRIM5-sensitive capsids promote assembly of TRIM5 lattice and subsequent activation of TRIM5-TAK1 signaling. This leads to IFN-b up-regulation and restriction of TRIM5-insensitive viruses including WT HIV and Sendai. We have now included a schematic explaining our model in Fig. 9.

- I wonder whether the impact on Nf-kB signaling is a common feature of that signaling pathways or unique for TRIM5a-dependent induction? The authors should include controls in the assays in Fig.1, e.g. overexpression of IKKa/b or other means to stimulate an Nf-kB response?

We performed a new experiment using the luciferase reporter assay that showed that knockout of BECN1, ATG7, and ULK1 had no impact on the ability of over-expressed TAK1 to stimulate

NF-kB, but did reduce the Nf-kB activation by TRIM5. This result shows two things: 1) that autophagy factors are not generally required for Nf-kB activation in this assay and that the effects are specific to TRIM5; and 2) that the impact of autophagy factor knockout appears to be focused on the step between TRIM5 and TAK1 in agreement with our model. These data are now presented in Figure 2D.

- In Fig. 2D, the IFNb levels induced by RhTRIM5 and HuTRIM5 are the same, however huTRIM5 has no effect on virus replication. Would that mean that the signaling of TRIM5a does not impact the virus?

We really appreciate this comment. It is very clear from our study and other published work that TRIM5 over-expression activates interferon b expression. It is also very clear that expression of rhesus, but not human, TRIM5 can restrict HIV. This raises the question, as asked by the reviewer, how relevant the interferon/TRIM5 signaling is to antiviral response. Our data demonstrate that the signaling that results from TRIM5 stimulation with a restricted retrovirus can trigger antiviral responses. We showed that these responses relied upon NF-kB and AP1 signaling (Fig. 7D, S4G). Knock-out of autophagy factors and chemical inhibition of autophagy blocked TRIM5 signaling and also reduced the antiviral impacts of priming in our assays. These data strongly indicate an importance of TRIM5 signaling in establishing antiviral states. However, we cannot at this time explain why TRIM5 over-expression and consequent signaling is insufficient to block viral infection. We hypothesize that TRIM5 over-expression is a poor surrogate for TRIM5 activation by virus, but we do not know precisely how these differ. We now address this question in the discussion and in the summary figure (new Figure 9).

- Please include TRIM5a KO in Fig. 3B and C.

We have repeated the experiments testing the role of autophagy factors – and now TRIM5 – in the ability of HIV-1 CA P90A to stimulate IFN-b expression. Our data show that TRIM5 knockout reduces IFN-b expression in mock infected cells and also prevents an IFN-b response to P90A infection. These data are found in Fig. 5B. We have removed the NLRP1 dataset (originally Fig. 3C).

- Fig. 6D and E: The IP levels of TAK1 and phosphor-TAK1 visually correlate with Input FLAG-TAK1 or GFP-TRIM5, please quantify these IPs and normalize to the respective controls, to strengthen the conclusion.

For both of the criticized co-IP datasets (now in Figure 8), we have provided quantitation from three experiments. We also replaced the data shown in panel E with a different experiment that more clearly shows the diminished interaction between TRIM5 and TAK1 in cells lacking BECN1 or ATG7. We no longer show phospho-TAK1 in these experiments since its abundance in the IP tended to correspond with its abundance in the input.

Reviewer #2: (No Response)

Reviewer #3: I offer a few minor suggestions for the authors to consider:
1) Potential candidates for the regulation of TAK1 activity by TRIM5a could be TAB2/TAB3. The authors should test in their experimental settings if TAB2/TAB3 levels and/or their interaction with TAK1 are altered by downregulating the expression of autophagy genes.

The reviewer is correct that several reports of TRIM5 impact TAB2 expression exist in the literature. A previous study (Tareen and Emerman 2010, PMID 21035162) indicated that TRIM5 expression can promote lysosomal degradation of TAB2. We subsequently showed (Kehl, Soos, et al PMID 31347268) that both TAK1 and TAB2 are subject to autophagic degradation. Based on these findings, autophagy might be expected to attenuate TRIM5-TAK1 signaling by degrading TAB2 (and/or TAK1). However, this is the opposite of what we report here, where the lack of autophagy proteins decreases signaling through the TRIM5-TAK1 axis.

2) It remains unclear if, besides autophagy proteins, the autophagic degradative activity is also required for the regulation of NF-kB signaling. It would be interesting to test if lysosomal inhibitors affect IFN induction by TRIM5a.

We agree with the reviewer, and so we tested whether inhibition of lysosomal proteolysis would impact the ability of TRIM5 to activate NF-kB in our luciferase reporter system. These data are now shown in Figure 3F. We saw that treatment of cells with lysosomal protease inhibitors results in ~20% reduction in NF-kB activity in cells expressing TRIM5. From this, we conclude that autophagic degradation is probably not the main mechanism underlying the actions of the autophagy machinery on TRIM5-TAK1 signaling, but our data do not exclude the possibility that some negative regulator of TRIM5-TAK1-NF-kB could be degraded by autophagy.

Part III – Minor Issues: Editorial and Data Presentation Modifications
Please use this section for editorial suggestions as well as relatively minor modifications of existing data that would enhance clarity.

Reviewer #1: - Please replace Fig. 2C with a more quantitative FACS assay

The high content imaging assays that we used in this study image and analyze a similar number of cells as is typically performed in flow cytometry-based assays. These data (now Figure 4A) show that THP-1 macrophage-like cells expressing rhesus TRIM5 are strongly protected (>10 fold) from infection with HIV-1 pseudovirus.

- Fig 5 C and D would benefit from a more transparent presentation of the data

In our study, we presented the results from the STR (secondary TRIM5 restriction) experiments in two ways. First, we show raw data from high content imaging experiments showing the percentage of cells that are infected as in Fig 5B (now Figure 7B). We also show a more distilled version of the data plotting "Relative restriction", which is the difference in the infection rate of un-primed versus primed cells. Figure 5C (now Figure 7C) shows the data from Figure 5B and additional biological replicates in the latter format. In response to the reviewer, we have now included raw data corresponding to the original figure 5D (now figure 7D). This new plot is now shown in Figure S4G.

- Fig 6 A has to be quantified: phosphor-Tak vs Tak levels

We replaced the experiment formerly shown in figure 6A with a slightly different approach to test the role of autophagy factors in TAK1 activation by TRIM5. This new data set and the corresponding quantitation is now shown in Figure 8A. As before, we see that the expression of TRIM5 expression (now with a C-terminal APEX2 tag that we used in most of the luciferase experiments) increases the relative abundance of phospho-TAK1 in WT cells but not in BECN1 or ATG7 knockout cells.

- Fig 6B and C, all the single overexpressions and stains need to be shown. Please quantify the co-localisation.

For these three-color confocal images, we now show single-color insets that demonstrate multiple structures positive for GFP-TAK1, TRIM5-HA, and either myc-tagged ULK1 or endogenous p62. Following deconvolution, these images have a horizontal resolution of ~130 nm which is technically in the super-resolution range. We do not provide quantitation of triple co-localization since such analysis (e.g. Pearson's or Mander's coefficients) are designed to measure correlation between two colors.

- Fig. 6G: This dataset is relatively weak, although it is explained why. However, are there any other mutants of TRIM5a which may be more suitable to show that autophagy is required for Nf-kB signaling?

Unfortunately we are not aware of other mutants that would perform suitably in these studies. Our previous work mapped TRIM5 domains required for interactions with autophagy factors ULK1, BECN1 and LGALS3, which we found to bind to the C terminal SPRY domain and also to the CC domain. Finer mapping of these interactions would be required to identify mutations that disrupt TRIM5-autophagy machinery interactions without disrupting the assembly of higher-order structures or TRIM5's enzymatic activity.

- Processing of signaling compounds may be important. But also degradation of NIK (and other negative regulators of Nf-kB signaling) as a major factor (besides DUBs), could be experimentally checked or addressed in the discussion.

We agree that autophagic degradation of multiple regulatory factors could be involved. In fact, our new data (Figure 3F) show that inhibition of lysosomal proteolysis with the inhibitors e64d and pepstatin A modestly attenuates the impact of TRIM5 expression on NF-kB. It is also possible that blocking autophagy increases the proteasomal degradation of a factor or factors that act to inhibit signaling through the TRIM5-TAK1 axis. While we did not specifically mention NIK, we do further emphasize the possible degradative role of negative factors in the figure legend detailing our model (new Figure 9).

- Can the authors include a model of their suggested mechanism?

In response to this suggestion, we have now included a schematic summary of our findings and model. This is presented in Figure 9A,B.

Reviewer #2: L139ff. It appears that BECN1, ATG7, ULK1 knockdowns/outs have a greater effect on NF-KB than AP-1. Is this true? If so what does it mean?

We also noticed that TRIM5-driven AP1 is less impacted than TRIM5-driven NF-kB. Both the NF-kB and AP1 pathways have multiple points of regulation downstream of TAK1. It is possible that autophagy factor knockout may also affect some of the positive or negative regulators of the NF-kB or AP1 pathways. These impacts could be either direct or indirect. The cumulative effect of autophagy factor depletion on these downstream regulatory factors could result in a level of autophagy dependency that differs between NF-kB and AP1. Since the focus of our study is TRIM5, we feel that detailing how other aspects of the NF-kB and AP1 pathways are impacted by autophagy is beyond the scope of this study.

L141. Requiring

This typo has been fixed.

L183ff. There appear to be differences between hu and rhTRIM5, especially in NLRP1 responses. Do you think these are important? I wonder whether it would be worth testing RBCC/PrySpry chimeras?

Other groups (e.g. Tareen and Emerman PMID 21035162) have previously reported that human and rhesus TRIM5 differ in the magnitude of their ability to induce signaling when over-expressed in cells. They also determined that the SPRY domain appears to be dispensable for TRIM5 to induce signaling. Whether the differences in over-expressed rhesus versus human proteins translate into biologically relevant differences is not clear.

L202ff. Is over-expression in some way similar to CA binding? Could it be that hexameric TRIM5 is needed for TAK1 activation and that this occurs at a certain rate naturally and is enhanced by over-expression or virus binding?

We believe that TRIM5 over-expression is a surrogate for CA binding, but likely an imperfect one. This is illustrated by the fact that while both CA binding and over-expression can promote some level of immune activation but that over-expression of human TRIM5 on its own is insufficient to protect cells against HIV infection. We are aware of a manuscript deposited in BioRxiv (Carter et al) that indicates the formation of TRIM5 hexamers in the absence of exogenous restricted CA.

No reference in text to Fig S3A

This has been fixed.

L455ff. Is the preparation of deltaLIR1/2 described here?

This plasmid was previously described and we now provide the reference.

Fig 2. What is Scr siRNA? Is Beclin1 siRNA the same as BECN1 siRNA in Fig 1?

We have re-labeled this figure. Scr was short for scrambled siRNA, which is not technically correct in this case. We now refer to this as non-targeting (NT) siRNA. We have also tried to replace all references to "Beclin 1" with the official name "BECN1" throughout the manuscript and figures to maintain uniformity.

Fig 4. In Fig 3A CrFK moi-3 gave c.30% infected cells in 4C CrFK a moi of 2 gave c.50% infection. Needs an explanation. Also the differences going from moi of 2 to 1 in Figs 4C,D

We apologize for accidentally mislabeling the figure legend for Fig 3A (now Fig. 5A) in our original submission. For this data set, we actually used a CrFK MOI of 1 (not 3). This has been corrected in the revised manuscript.

Fig 6A. Does ATG7 really increase amount of p-TAK1 in GFP control?

We replaced the experiment formerly shown in figure 6A with a slightly different approach to test the role of autophagy factors in TAK1 activation by TRIM5. This new data set and the corresponding quantitation is now shown in Figure 8A. As before, we see that the expression of TRIM5 expression (now with a C-terminal APEX2 tag that we used in most of the luciferase experiments) increases the relative abundance of phospho-TAK1 in WT cells but not in BECN1 or ATG7 knockout cells. As shown in the graph, we did not see an increase in pTAK1 in the control cells (now transfected with APEX2 instead of GFP).

Fig 6B, C. Very hard to make out important details

We now provide zoomed-in insets with single-color images. These more clearly show the triple co-localization between TRIM5, TAK1, and autophagy proteins.

Fig 6E. Is GFP-TRIM supposed to go down in IP but not in input?

We provided a different experiment and quantitation of the abundance of immunoprecipitated GFP-TRIM5 relative to GFP-TRIM5 in the input. The data show that there is less interaction between FLAG-TAK1 and GFP-TRIM5 in cells lacking ATG7 or BECN1. These data are now shown in Figure 8E.

Fig 6F. Why are there two sets of FLAG-TAK1 samples?

One of the FLAG-TAK1 blots showed immunoprecipitated FLAG-TAK1 while the other showed the FLAG-TAK1 in whole cell lysate (input). We adjusted the figure to make this more clear. We also added a plot quantitating the relative abundance of phospho-TAK1. This is now shown in Fig. 8F.

Fig S3A. Is it fair to say that on a per cell basis HIV P90A is more efficient that poly(I:C)?

We do not feel that we have sufficient data to make this claim. Under the conditions tested, P90A yielded comparable IFN-B up-regulation as did poly(I:C). However, we have observed that the level of IFN-B mRNA induction is dependent on the duration of the stimulation period with initial robust induction followed by declining IFN-B expression. As we did not test multiple time points following induction with poly(I:C) or P90A, it is possible that the two treatments lead to IFN-B induction with different kinetics. It does appear that P90A priming was more effective at blocking Sendai virus infection than was poly(I:C) using our experimental conditions.

Fig S5B. See labels GFP-RhTRIM5 and GFP-TRIM5. Is the Rh a mistake? Are all these experiments done with wt and deleted human TRIM5?

As indicated in the methods, the GFP-tagged TRIM5 expression plasmids used in the study were based on rhesus TRIM5. The labeling has been amended for uniformity.

Reviewer #3: None

PLOS authors have the option to publish the peer review history of their article (what does this mean?). If published, this will include your full peer review and any attached files.

If you choose “no”, your identity will remain anonymous but your review may still be made public.

Do you want your identity to be public for this peer review? For information about this choice, including consent withdrawal, please see our Privacy Policy.

Reviewer #1: No

Reviewer #2: No

Reviewer #3: No

Figure Files:

While revising your submission, please upload your figure files to the Preflight Analysis and Conversion Engine (PACE) digital diagnostic tool, https://pacev2.apexcovantage.com. PACE helps ensure that figures meet PLOS requirements. To use PACE, you must first register as a user. Then, login and navigate to the UPLOAD tab, where you will find detailed instructions on how to use the tool. If you encounter any issues or have any questions when using PACE, please email us at figures@plos.org.

Data Requirements:

Please note that, as a condition of publication, PLOS' data policy requires that you make available all data used to draw the conclusions outlined in your manuscript. Data must be deposited in an appropriate repository, included within the body of the manuscript, or uploaded as supporting information. This includes all numerical values that were used to generate graphs, histograms etc.. For an example see here on PLOS Biology: http://www.plosbiology.org/article/info%3Adoi%2F10.1371%2Fjournal.pbio.1001908#s5.

Reproducibility:

To enhance the reproducibility of your results, PLOS recommends that you deposit laboratory protocols in protocols.io, where a protocol can be assigned its own identifier (DOI) such that it can be cited independently in the future. For instructions see http://journals.plos.org/plospathogens/s/submission-guidelines#loc-materials-and-methods

In compliance with data protection regulations, you may request that we remove your personal registration details at any time. (Remove my information/details). Please contact the publication office if you have any questions.
